# A three tier rapid mass programming method

**DOI:** 10.1016/j.mex.2019.10.003

**Published:** 2019-10-07

**Authors:** Tyler Steane, Michael Trifilo, Christopher Rogash, P.J. Radcliffe

**Affiliations:** School of Engineering, RMIT University, Melbourne, Australia

**Keywords:** IoT, WSN, Testbed, Networking, Protocol testing

## Abstract

Various research activities require the programming of a large numbers of devices. This programming can be difficult to co-ordinate and organise, and requires considerable labour time. These issues often mean that testing on real hardware is abandoned or taken only to small scale implementation thus limiting the real-world findings. The method described in this paper adopts a three-tiered approach to programming large numbers of devices. Tier 1 is comprised of a single Master Controller which is networked to individual tower modules, these towers form the final 2 tiers with the Local Controller as tier 2 and up-to 15 target devices forming tier 3. The Master Controller co-ordinates and distributes the code for each device to the Local Controller which then programs the target devices. In the domain of networking this allows for:

• Large networks of varied protocols to be programmed quickly, since towers are programmed in parallel, additional towers don’t extend programming times.
• Distributed networks are possible since towers are controlled over Ethernet.
• Dramatically reduced labour time and defect rates due to human error in setting up devices.
• This paper presents the implementation of this method for IoT Networking research with ESP-01 Target devices.

## Graphical abstract

**Figure:**
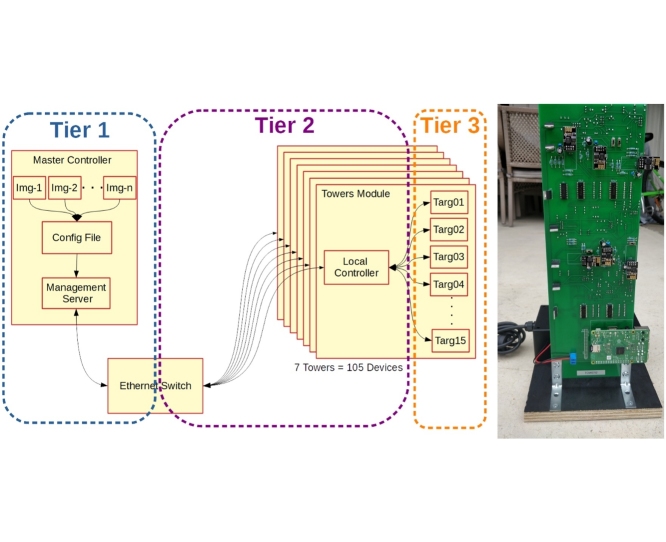

**Specifications Table**

**Table:** 

Subject Area:	*Engineering*
More specific subject area:	*Internet of things Protocol design and testing.*
Method name:	*N/A*
Name and reference of original method:	*N/A*
	*Most common method is trivial and ubiquitous, wired programming*
Resource availability:	https://github.com/TylerSteane/three-tier-ESP-testbed-Tower

## Method details

The aim of the proposed methodology is to enable rapid mass programming of physical Target devices. This methodology has been specifically implemented to support research into IoT networks built from ESP8266 type devices, using the ESP-01 modules, but could just as easily be applied to any other application with large numbers of target devices that need programming. All materials needed to reproduce the test-bed implementation of this methodology can be found here [1], including schematics, PCB layouts, and code.

This method implements a three-tiered approach to programming devices. The first tier is the Master Controller, while the second tier is comprised of multiple Local Controllers embedded with the third and final tier which is the Target devices to be programmed or flashed. Tiers 1 and 2 are connected via Ethernet, this can be as simple as using a network switch (as presented here) but could also be distributed more broadly across a larger area using a more complex network setup. Tiers 2 and 3 are plugged into sockets mounted on PCBs to form tower modules with one Tier 2 Local Controller to several Tier 3 target devices.

Different test applications will require different network configurations, as has been mentioned the tower modules can be distributed around a wide area representing clusters of devices. For this reason, when target devices are plugged into the tower modules they can be mounted in various orientations in the X, Y and Z plane. However, when individually scattered devices are required for testing, target devices can be programmed on the tower modules for convenient programming and then removed and connected to other supporting hardware for individual scattering.

In the case of our IoT implementation, the Master controller is run on a PC while Local Controllers are run on multiple Raspberry Pis connected to the Target device ESPs via a single serial port which is multiplexed to handle up to 15 IoT devices.

**Fig. 1:**
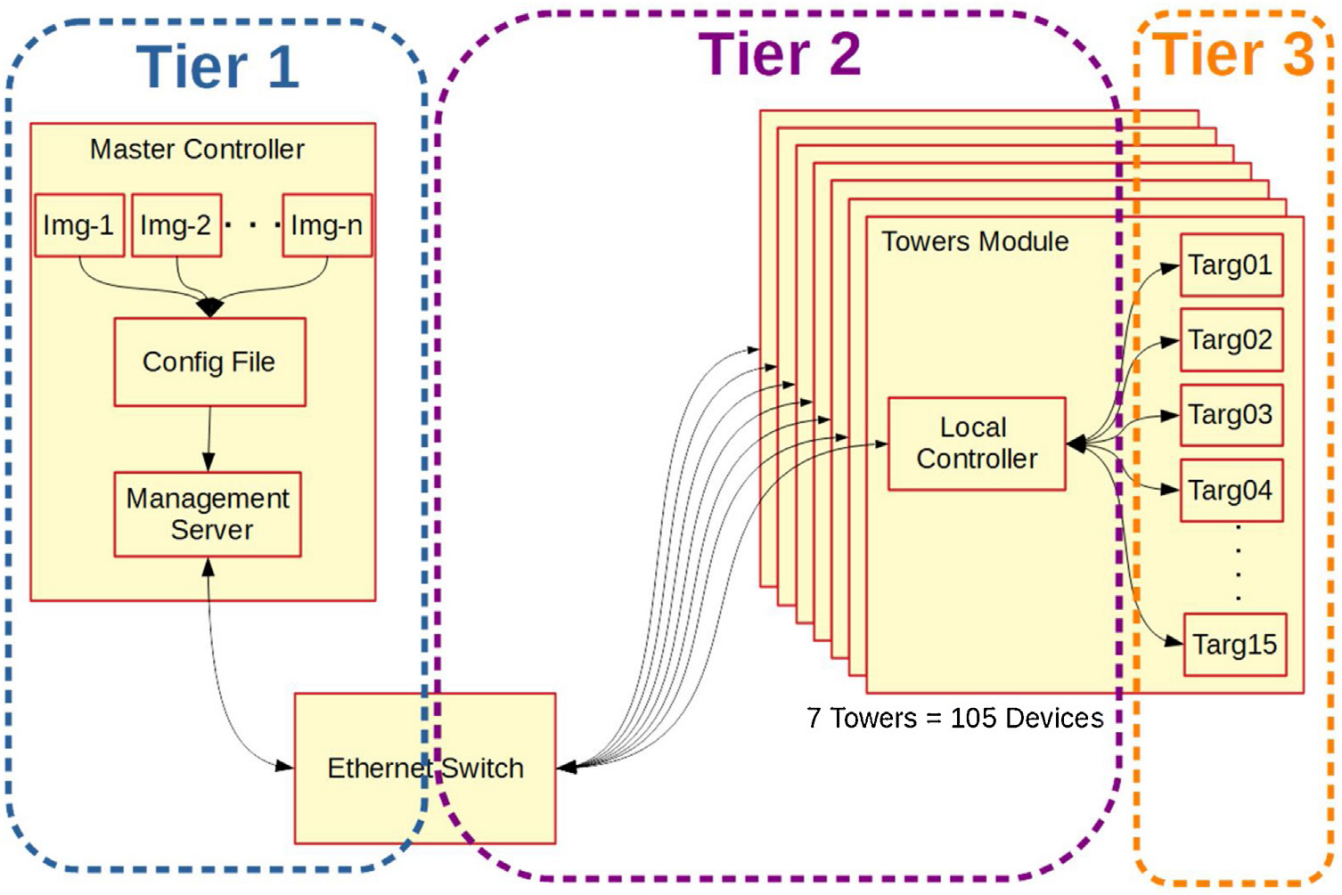
Simplified Block Diagram of Methodology's Test-bed.

Once the tiers are setup and configured, programming is a fairly simple task, summarised below and in Fig. 1:

1 Image files are prepared for the target devices
2 A simple configuration file is prepared, detailing which images are to be distributed to which devices.
3 These files are then feed into the Master Controller which will distribute programming commands and files to all connected Local Controllers.
4 Local Controllers then begin programming each of the target devices one at a time.
5 Once a device is programmed it is then restarted and its status logged.

To understand the operation of this method, it is helpful to first understand how the test-bed’s architecture achieves its functionality.

**Fig. 2:**
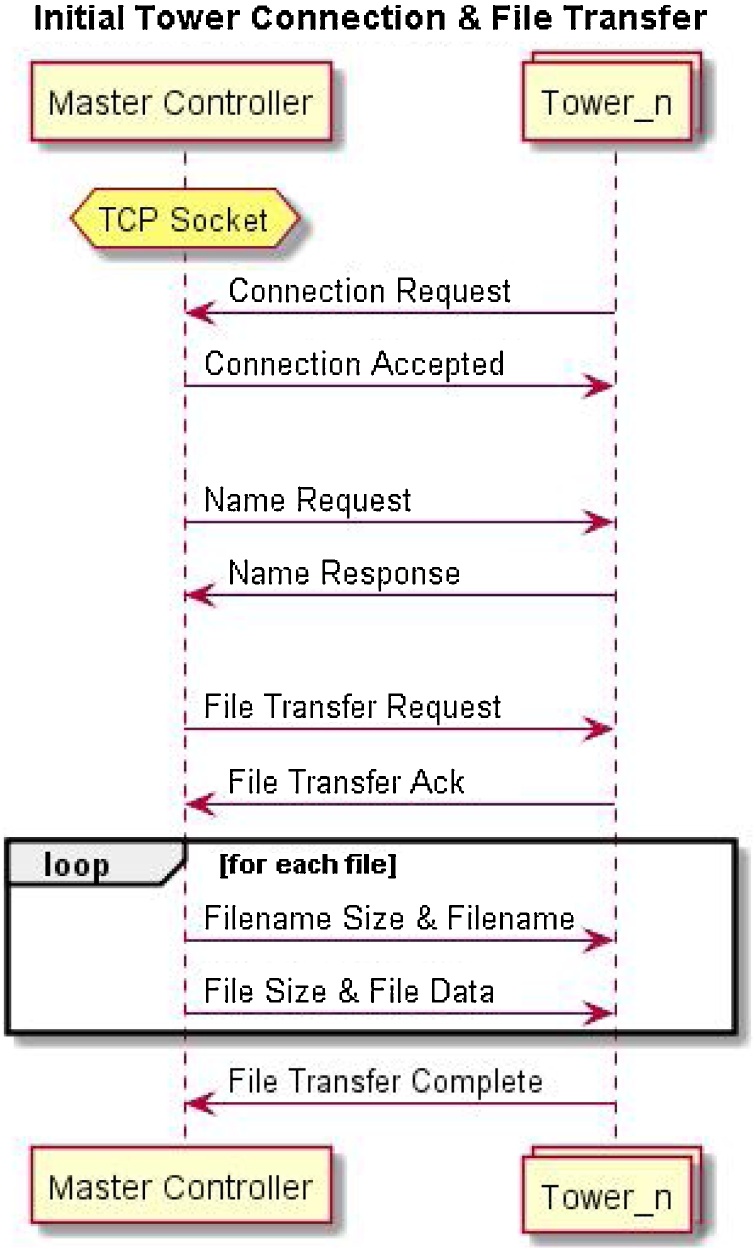
Sequence Diagram for initial connection of Master and Local Controllers.

**The Master Controller** is run as a Python script, Python offers socket libraries to build up a TCP interface. Multi-threading is used to handle connections to towers, a new thread is created upon receiving a request for connection from a tower. Once a tower is connected, the Master Controller will then begin transferring all the different, pre-compiled image files to be programmed on the target devices. If all devices are being programmed with the same image only one file will be transferred. The controller begins by first sending the filename and size, which can be used to validate the file transfer, followed by the file itself. This process is summarised in Fig. 2. Once the controller has transferred all the files, it begins sending commands to the Local Controller to program each target device and logs the results.

**Fig. 3:**
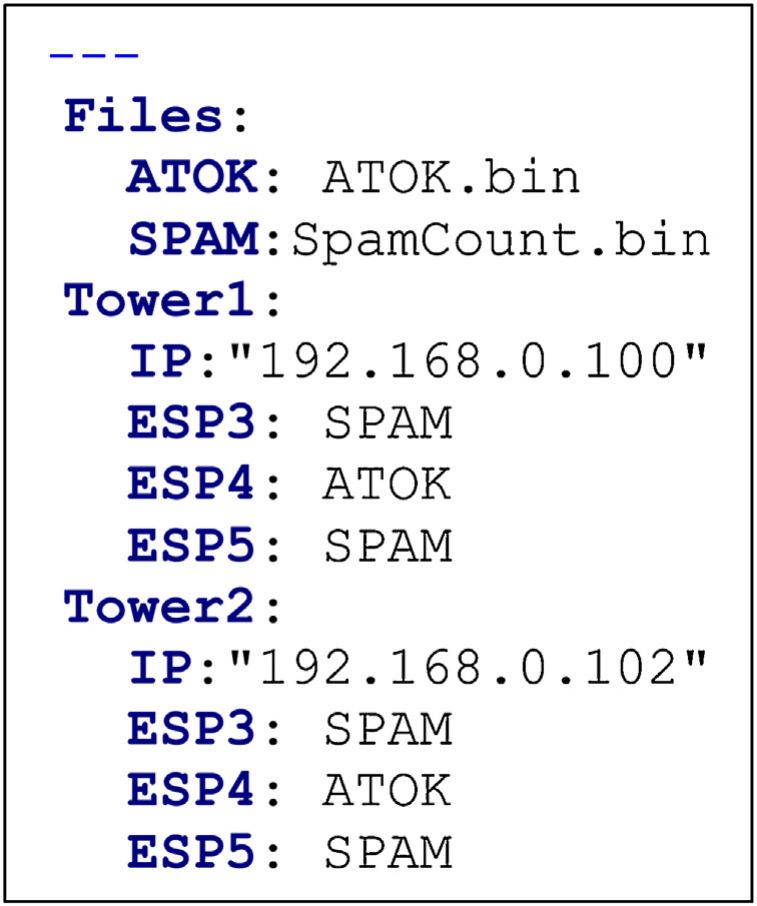
An example of a YAML configuration file for programming two different images to 6 target devices on 2 towers.

**The Configuration File** uses the YAML language to create a human-readable configuration that is easy to write and interpret. Fig. 3 shows an example of a configuration file. Image files are specified at the top of the file and assigned an alias which can be repeated below more easily. The rest of the file is simply a list of towers and the target devices with the assigned image. Note that not all target devices need to be specified and only those specified will be programmed.

Future work will aim to further simplify these files by allowing for key words such as “ALL”, “EVEN”, and “ODD” to assign images to all devices on a tower more quickly. Further support would also be added to assist in validation and error correction in these files.

**Fig. 4:**
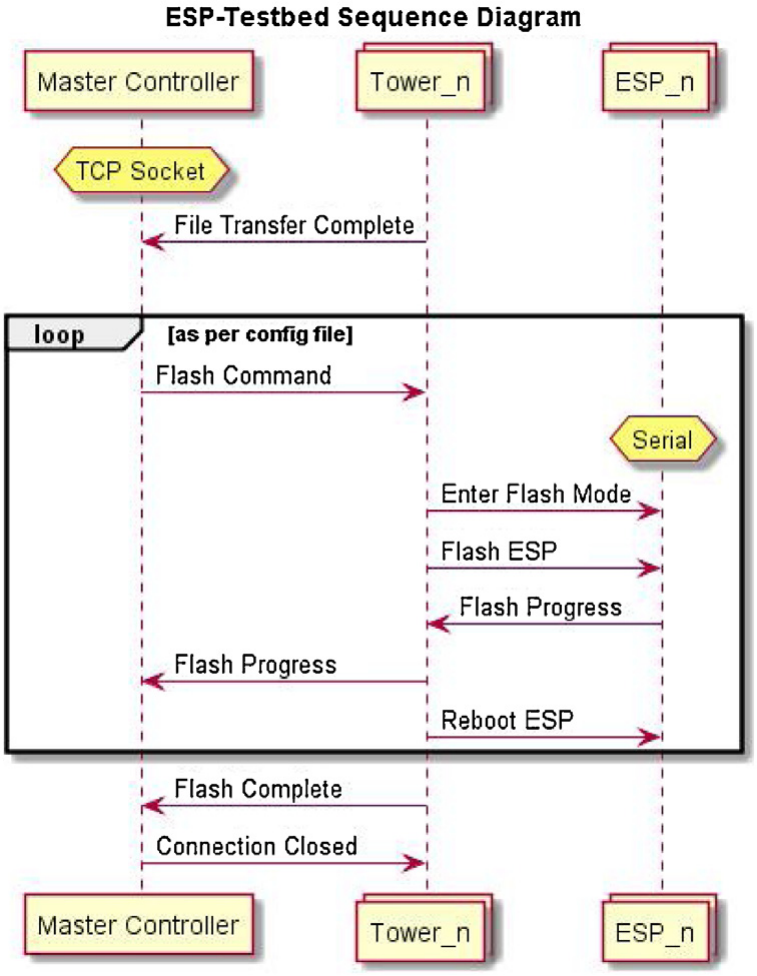
Sequence Diagram for programming, “Flashing” of ESP target device.

**The Local Controller** similar to the Master Controller, is a python script that runs on a Raspberry Pi connected to each tower. It is connected to the Master Controller via the Raspberry Pi’s Ethernet port, and the script will automatically attempt to connect to the controller on start-up. As described above, the Local Controller receives files and programming commands from the master controller, it then uses the ESPtool to program the target devices as detailed in Fig. 4. The Local Controller uses 2 Serial Port pins and 2 GPIO pins to program target devices. These 4 pins are multiplexed using analogue switches for bi-directional connections meaning no pre-configuration of Target devices is required. This approach allows for greater flexibility in the test-bed allowing for logging of data back from the target devices serial lines after devices are programmed and running. In total 8 GPIO pins from the Raspberry Pi are used, 4 for data and 4 to select the correct target device before programming.

**Target Devices** this method has focused on the programming of ESP8266 family Chips and specifically the ESP-01 modules. However, the PCB design could easily be adapted to program other ESP modules, and the schematic and method could be adapted to programming other devices.

**Fig. 5:**
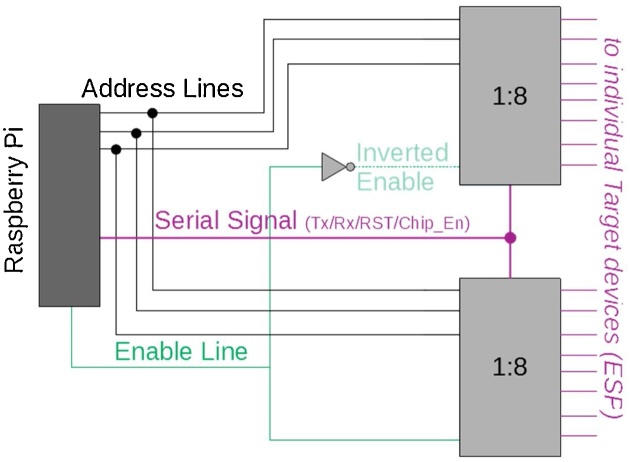
Multiplex configuration used for each line of the serial port connecting the Local controller to Target Devices, using 2 1:8 Mux devices and an enable line to select between the two Muxs.

**Tower Architecture for ESP unit** is centred around a custom PCB with the multiplexing circuitry, shown in Fig. 5, to connect the Target devices to the local controller, a Raspberry Pi. This circuit uses 4 pins on the ESP target devices, Tx, Rx, Chip_enable and RST, to program or ‘flash’ the target devices one at a time.

As has been mentioned, the tower module could easily be distributed around a large network and this is to facilitate the testing of the Programmed protocols on the target devices. To this end 3 other considerations have gone into the design of the towers. Firstly, the towers have a robust power supply capable of powering the Raspberry Pi Controller as well as all 15 devices at once even at their highest loads. This power circuitry maintains 3.3 V to each ESP as well as 5 V to the Raspberry Pi. Secondly, the layout of the towers allows for a variety of orientations for the target devices and with the addition of 90° daughter board devices can be oriented in x, y and z planes to accommodate testing sensitive to physical orientation. Thirdly, the Raspberry Pi and the bi-directional multiplexing circuit allow for the logging of data coming back from the target devices serial lines, allowing for detailed performance analysis of protocols in a physical implementation.

This tested architecture allows any number of pre-compiled image files to be programmed to any number of target devices quickly and easily. Image files are assigned to devices in the configuration file which is supplied to the management PC that will distribute the images to the relevant towers and then begin supplying programming commands to the towers. The Local Controllers on the towers then select the appropriate device using the multiplexing circuit and flash the image file to the device.

## Implementation and usage

This methodology can be applied to any situation where multiple devices need to be programmed but our work has focused on applying it to IoT devices. The following procedures will demonstrate how this specific application can be implemented and will give an idea of what will be required for different applications.

### Configuring a tower controller

The ESP programming version of the three-tiered programmer used a Raspberry Pi 3 model B+ as the controller. The following is the procedure to set up this controller:

**Step 1**: Install Raspbian to a micro SD card as per the Raspberry Pi documentation.

When a Raspberry Pi is started for the first time, the Welcome to Raspberry Pi application will pop up and guide the user through the initial setup. Follow this as advised in the Rasbian documentation.

**Step 2**: It’s vital to configure the correct System and Interface settings if this step isn't followed correctly the Pi script won’t function correctly. Under Menu -> Preferences select the Raspberry Pi Configuration settings.

In the System tab, change the following settings:

• Change the Hostname to something unique. In our prototype environment, we used the relevant tower number i.e. "Tower1", "Tower2"
• Resolution: 1920 × 1080 (Helpful for VNC Debugging)

In the Interfaces tab, change the following settings:

• SSH: Enable (Recommended for debugging & remote configuration)
• VNC: Enable (Recommended for debugging & remote configuration)
• Serial Port: Enable (IMPORTANT)
• Serial Console: Disable (IMPORTANT)
• Reboot the Raspberry Pi

**Step 3**: Configure Network Settings. As the ESP-01 modules can be used for WiFi testing it is recommended that the Towers are hardwired to the Management network. In our prototype system, each device in the network is set with a static IP. Configure all Towers and the Management PC with unique static IP addresses. Note that the default configuration for the Raspberry Pi is to connect to 192.168.0.100, this should be assigned to the Master Controller. So, for a 3 Tower network the IP assignment might look like:

• Network Address: 192.168.0.0/24
• Management PC IP: 192.168.0.100
• Tower1: 192.168.0.101
• Tower2: 192.168.0.102
• Tower3: 192.168.0.103

**Step 4**: Install software. This can be done in the command line with the following commands.

• Python should be installed by default.
• Install the Serial library for python:

 pip install pyserial

• Install xterm for better support to run the Local Controller on start-up:

 sudo apt-get install xterm

• Install the ESPTool for programming the ESPs:

git clone https://github.com/espressif/esptool.git

**Step 5:** Configure the Local Controller to run on start-up,

This is done by editing the ‘autostart’ file:

 sudo nano/home/pi/.config/lxsession/LXDE-pi/autostart

Before the line:

 @xscreensaver -no-splash

**Fig. 6:**
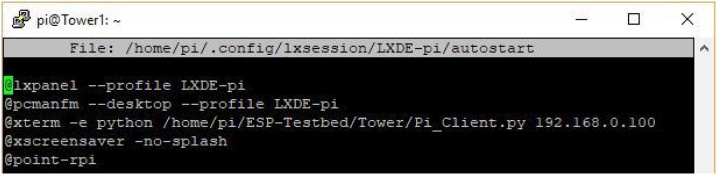
Auto start file after being edited to run Local Controller on start-up.

Add the following line (see Fig. 6):

 @xterm -e /usr/bin/python /home/pi/ESP-Testbed/Tower/Pi_Client.py 192.168.0.100

Finally save the file and reboot the Raspberry Pi.

### Regular operation

Regular operation is very simple and can be done from the Master Controller, assuming all towers are set up and configured correctly and that all target devices are installed.

1 First all Image files should be pre-compiled, this can be done in an IDE like the Arduino IDE.
2 The configuration file needs to be written to define which files are to be programmed onto which devices.
3 The management PC is then run with the following command:

 ./mgmt.py -configfile.YAML

**Fig. 7:**
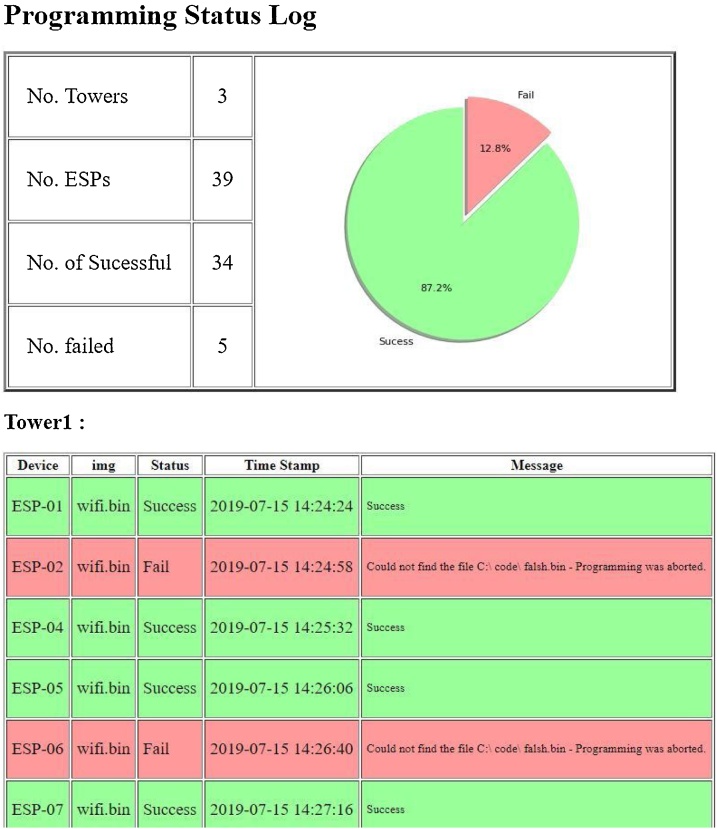
Log Status after programming 39 Target devices with 5 known faulty devices demonstrating the diagnostic capabilities of the logging.

4 Once this process is completed, the results of programming can be seen in the log file (see Fig. 7 bellow for example.):

 ./Mgmt_PC/Log.html

5 The target devices can now be run and tested.

Experience has shown that it takes some time to create the first configuration file, but subsequent configuration files are very easy to create.

## Performance and validation

In order to test and validate this method, we programmed 39 target devices. This use-case aimed to evaluate the number of devices that could easily be accommodated on a typical consumer WiFi router. This testing was done with 3 Towers Modules each with 13 target ESP8266-01 devices, 39 target devices in total. Each target device was loaded with a simple program that would join the device to a predefined wireless network. The log results in Fig. 7 show the results were 5 of the 39 devices were known to be faulty and failed to load properly. This demonstrates the diagnostic capabilities of the logging. The known faulty devices were then replaced with working devices for a 100% successful programming of all 39 target devices. Then target devices were progressively turned on and the network load tested.

**Fig. 8:**
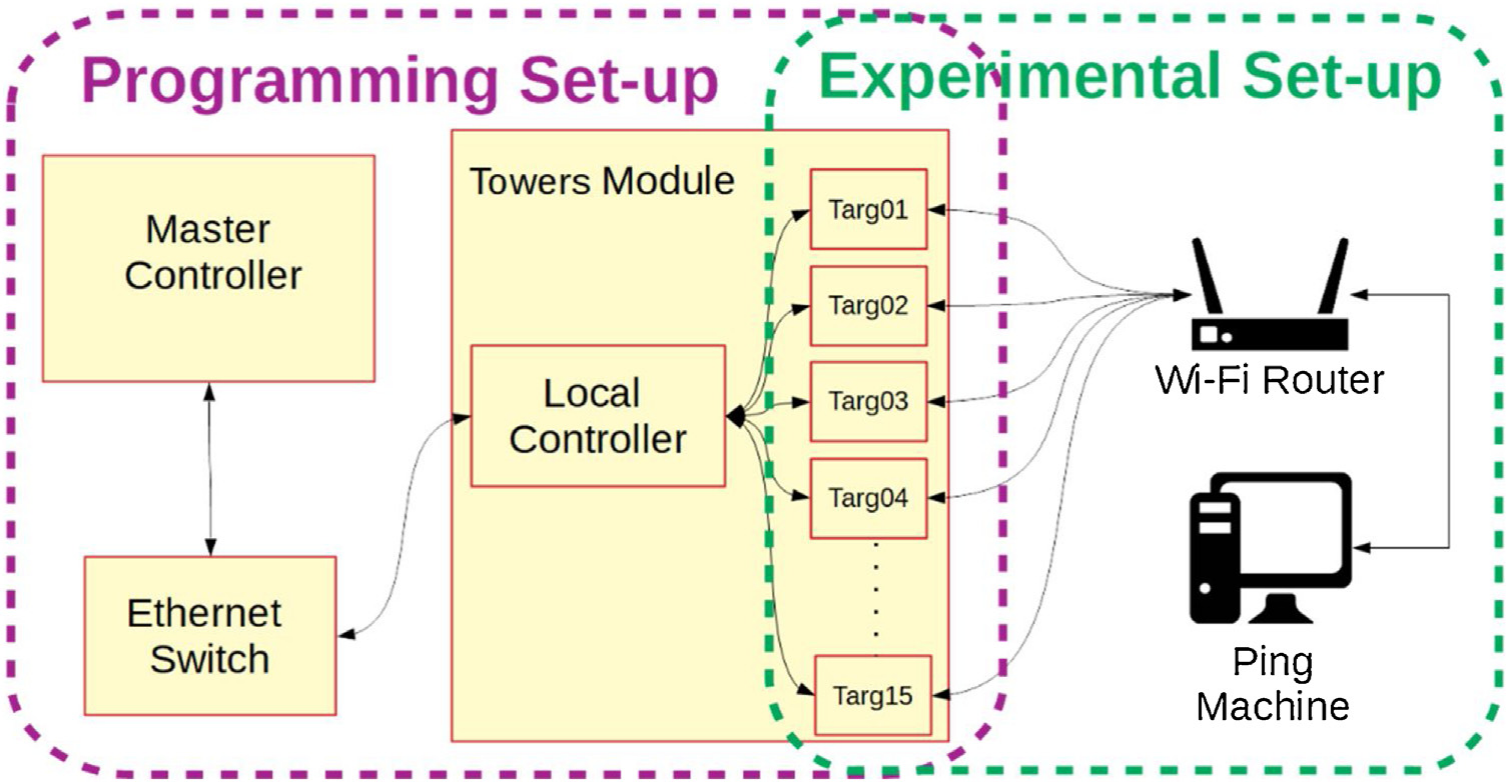
Programming and experimental set-up used to implement and validate methodology.

To test the capacity of a domestic WiFi network each device was sent a ping packet from a PC connected to an Ethernet port of the same WiFi router which was connected to the target devices, see Fig. 8 for experimental set-up. These packets were sent to each target device one at a time, to check for connectivity. Each device was pinged with a 500 ms timeout with up to 10 retries if a request timed out. This test was run as more and more devices were added to the network. Devices where added 3 at a time, one on each Tower module. The test results where then analysed to see the success rate of ping requests.

**Fig. 9:**
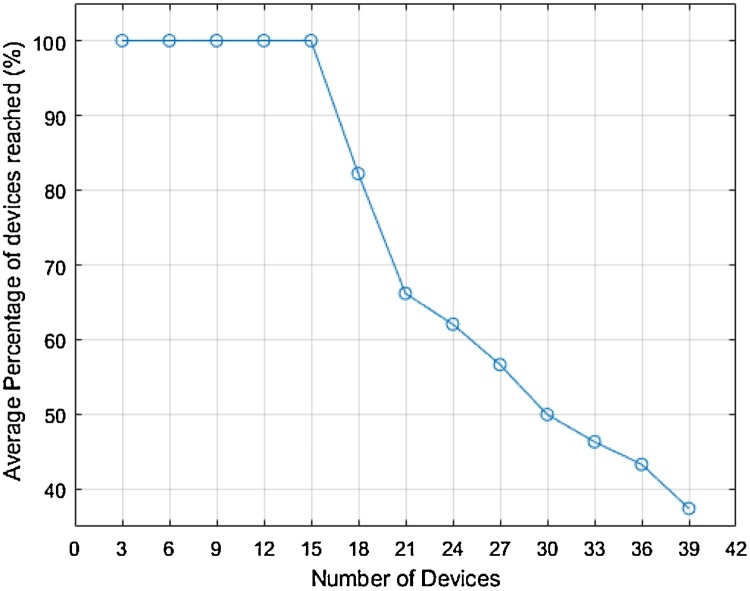
Percentage of devices reached as the network load is increased.

The results are shown in Fig. 9, each test pinged all connected devices one after the other and repeated 10 times to minimise anomalies. The WiFi router used is was a D-Link DIR-635, and towers were placed in a domestic setting in different directions and distances (∼2, 3 and 5 m away) from the router. Testing was run from a machine connected to the network via Ethernet.

The results in Fig. 9 show that after 15 devices additional devices begin to create issues on the network and not all devices can be reached. This is not a result of new devices not being acknowledged as new devices are usually accessible. The downward trend in the percentage of reachable devices is due to the increasing number of devices as the D-link router seems to support only 15 simultaneous wireless devices. Testing with an enterprise quality high capacity WiFi router, the Unifi UAP-AC-Pro, had no problems handing all 39 devices, and more, thus proving that the domestic WiFi router is limited in its capacity.

This testing was able to be carried out reasonably easily thanks to the methodology implemented. It was a simple task to program 39 devices, and then turn on the required number of devices using the switches next to each device on the tower modules. All devices were programmed in 7 min 11 s when all devices where properly working. The co-ordination of this method meant all devices where programmed without missing one and without the boredom associated with dull repetition, thus minimising the potential for human error and saving a lot of time.

## Additional Information

### Background

Programming large numbers of devices poses several key challenges:

• Organisation of which devices have been programmed, and which are still waiting.
• Co-ordinate which code and configuration data is programmed into each device.
• Time taken to program all devices.
• Labour time to program many devices, and to rectify mistakes.

These challenges are not easily overcome thus large scale physical implementation of a networking system is often abandoned. Research focuses instead on simulation and perhaps the results from small scale physical implementations. In our experience of network research, large scale implementation always finds significant effects that are not evident otherwise, thus large-scale implementation is imperative for research to be effective. Of these factors, labour time is perhaps the most significant particularly when some devices have different code or configuration data. Time is even more of an issue when there are multiple tests each with different code or configuration data.

Research into IoT has created a niche area for device programming. Small prototypes can manage with simple manual programming techniques, and large scale commercial or industrial applications can afford the overhead of more resource intensive automated programming methods. Research applications often require more awkward mid-sized networks which require complex configuration management, are too big for manual methods but not large enough for more demanding automated methods. The method described in this paper overcomes all these problems and provides a niche solution for research scale networks.

Currently, for networking applications like IoT or WSN the two main options currently available for programming devices are simple wired programming and over the air programming. Wired programming is a one device at a time process which is very slow and requires a lot of effort to keep track of more than a few devices and which version of the code they are running, it is often used for small scale network testing [2,3] Wired programming is often preferred for simple prototyping as the network is very small often only a one or two devices. When it comes time to test a larger network many will opt for models and simulations [4,5] but setting this up can be as involved, or even more so, than the protocols being investigated and the cost for specialised software can be prohibitive. Furthermore, this approach can easily miss affects and phenomena not predicted by the researcher, so for this reason many still prefer real-world testing on hardware but forgo large scale testing [3,6,7]. Many of these papers offer little or no discussion of the methodology used to program their devices.

The Wireless approach of ‘over-the-air’ programming can save a lot of time and can even improve some of the co-ordination and organisation of devices. However, it requires devices to be pre-loaded with supporting firmware which can take up significant space on constrained hardware like ESP-01’s. If the devices are not pre-loaded with supporting firmware then the slow and painstaking wired method must again be used if only once to setup everything. Additionally, memory must be set aside to store this firmware as well as room for the current image and any future image [8,9]. Memory requirements may be slightly reduced by using delta updates [[10], [11], [12]] but this is only helpful when modifying code but not completely changing applications. These memory requirements become increasingly problematic for constrained applications where devices have lower power and reduced storage so memory cannot so easily be set aside for such applications. Furthermore, constrained applications often implement slower memory modules to reduce over-all costs, this can often be done without compromising regular performance, but it does dramatically increase programming times especially if a one-at-a-time approach is taken.

Over-the-air (OTA) programming makes sense when all devices have the same code. When devices have different configurations, as is the typical case for research, then devices must be programmed one after the other which can take a long time for a large network.

Our methodology was motivated by testing network protocols for Home Automation where we anticipate high contention for the wireless medium. Here simulation is too expensive and real-world data is extremely valuable, but programming low cost simple ESP8266 chips like the ESP-01 module is a difficult process to manage for even 5 devices. Thus, the solution outlined in this paper has allowed us to scale our findings for 5 devices to 50+ devices.

Overall this approach has been essential to our IoT research and has uncovered some unexpected results. For example, many consumer home routers can only practically support 15-20 simultaneous WiFi connections. This is a real problem for a home with many IoT devices and requires a total rethink of how to implement an IoT based home or business.
